# Recognition of risk and prevention in safeguarding of children and young people: a mapping review and component analysis of service development interventions aimed at health and social care professionals

**DOI:** 10.1186/s12913-021-07257-8

**Published:** 2021-11-17

**Authors:** Duncan Chambers, Anna Cantrell, Andrew Booth

**Affiliations:** School of Health and Related Research (ScHARR), University of Sheffield, Sheffield, UK

**Keywords:** Safeguarding, Mapping review, Component analysis, Inter-professional working, Social care, Child abuse

## Abstract

**Background:**

The term ‘safeguarding’ covers the protection of health, wellbeing and human rights. Effective safeguarding enables people (particularly children, young adults and other vulnerable people) to live free from fear of abuse, harm or neglect. The UK Children Act 2004 required key agencies, including health and social care providers, to consider the need to safeguard children and promote their welfare. Within a larger evidence synthesis project, we sought to identify and map service development interventions (excluding provision of training) aimed at improving awareness of safeguarding and identifying at-risk children and young people in health and social care settings.

**Methods:**

We searched fourteen health and social care databases from 2004 (date of Children Act) to October 2019 and updated the review via a citation search in March 2021.

Studies of any design were eligible if they described or evaluated an intervention (other than training) aimed at health or social care professionals in the United Kingdom and designed to improve recognition of risk in the context of safeguarding children and young people. Studies with no intervention (e.g. qualitative studies) were included to explain why interventions work or fail to work. Included studies were summarised using narrative synthesis. Risk of bias of included studies and overall strength of evidence were assessed using standard methods. We used a 5-item checklist (“TIDieR-Lite”) to map intervention components.

**Results:**

Thirty-nine publications were included, of which 31 dealt with service developments, six with use of data and two with other initiatives. Promising service development initiatives include liaison nurses, assessment clinics, secondment, joint protocols and a ‘hub and spoke’ model. Initiatives involving use of routine data appeared promising and unlikely to generate significant additional costs. However, the quality of the evidence was generally low, with a shortage of controlled and long-term studies.

**Conclusions:**

Health and social care services wishing to improve awareness of child safeguarding issues may benefit from looking beyond high-quality training provision. Future research should focus on service-relevant outcomes and ensure the active involvement of young people and their families/carers.

**Supplementary Information:**

The online version contains supplementary material available at 10.1186/s12913-021-07257-8.

## Background

The term ‘safeguarding’ refers to measures designed to protect health, wellbeing and human rights, allowing people (especially children, young people and vulnerable adults) to live without fear of abuse, harm or neglect. The term is primarily used in the UK and Ireland, although the underlying concept is relevant to all health and care systems. The UK Children Act 2004 placed a responsibility on key agencies, including those in health and social care, to consider the need to safeguard children and promote their welfare. It follows that health and social care professionals at all levels need to be aware of safeguarding issues and procedures, although the amount and type of involvement with safeguarding will vary widely between professional groups.

The primary method of promoting safeguarding awareness is through provision of appropriate training, and various risk assessment tools and scales are available to health and social care professionals. However, broader organisational and cultural factors may also help or hinder people in recognising risk of abuse and taking appropriate action. Examples include co-operation between different organisations and professional groups, particularly at the interface of health and social care, and the use of information and data to promote safeguarding.

This paper presents and analyses data from a broader mapping review of research evidence on interventions to promote child safeguarding awareness in health and social care settings [1]. For this review, we aimed to identify organisational interventions and initiatives aimed at health and social care professionals that extended beyond the provision of training. The resulting narrative synthesis should be of value to research commissioners and decision-makers in health, social care and integrated care systems.

## Methods

Methods are reported in full in the technical report [1]. Briefly, the research was carried out in two stages. We systematically retrieved and coded UK research and policy documents to gain a contemporary picture of safeguarding issues and practice. Similar methods of searching and study selection were used for both stages. We undertook quality assessment of each primary UK study that reported a recognised study design.

We searched fourteen health and social care databases (ASSIA - Applied Social Sciences Index and Abstracts, CINAHL - Cumulative Index to Nursing and Allied Health Literature, Cochrane Database of Systematic Reviews, Cochrane Central Register of Controlled Trials, HMIC - Health Management Information Consortium, IBSS - International Bibliography of the Social Sciences, MEDLINE, PsycINFO, Sociological Abstracts, Social Care Online, Social Policy and Practice, Social Services Abstracts, Social Sciences Citation Index, and Social Work Abstracts from 2004 (date of Children Act) to October 2019. Citation tracking of the included national policy and guidance documents was conducted on Google Scholar. Searches for UK grey literature were conducted within the main database searches given that Social Care Online and Social Policy and Practice index grey literature. We updated the review in March 2021 by performing a citation search of all the originally included studies through Google Scholar.

Search results were uploaded to EPPI-Reviewer 4 (Evidence for Policy and Practice Information and Co-ordinating Centre, University of London, London, UK) for title and abstract screening. Screening was performed by a team of three reviewers. Individual records were screened by one team member, with a 10% sample being checked by a second reviewer for accuracy and consistency.

To be included in the systematic review, studies had to meet the following inclusion criteria:

- **Population** – Children and young adults (aged up to 18) and/or other service users (family members or other carers) in health and social care settings.
- **Intervention** - Interventions (other than training and awareness raising) aimed at health and social care professionals looking after children and young adults (aged up to 18) in health and social care settings and aimed at improving recognition of children at risk of physical, sexual or emotional abuse or neglect. Eligible interventions included, but were not limited to, new service models and job roles, and initiatives to improve the use of routinely collected data. Interventions that had training as the exclusive or main component were excluded.
- **Outcomes** – Improved knowledge and understanding of (risk factors for) abuse among practitioners. Improved rates of early identification of possible abuse. Qualitative outcomes, including feasibility and acceptability of interventions to professionals and young people. Any reported data on costs, resource use or cost-effectiveness. Other outcomes of interest included explanatory factors for why interventions are thought to work and findings of relevant cultural/organisational studies.
- **Comparator** – no intervention; comparisons with practice as usual were eligible for inclusion.
- **Study design** – we included primary literature from the UK (any design either quantitative or qualitative, including local service evaluations that met the eligibility criteria and contained relevant empirical data).
- **Other limitations** – For inclusion publications were required to be written in the English language and published since 2004 (the date of the Children Act).

Full papers were reviewed for all references that appeared to meet the inclusion criteria. Screening of full texts followed a similar process to that for title and abstract screening. Queries were resolved by discussion. Systematic and non-systematic reviews were coded for separate analysis.

Data extraction (coding) was completed in EPPI-Reviewer 4. Data from included studies comprised study design, intervention/initiative (where applicable), population/setting, results and key limitations. We extracted details from policy/guidance documents using a separate purpose-designed form. Data extracted were based in part on a safeguarding checklist produced by the National Society for the Prevention of Cruelty to Children (https://learning.nspcc.org.uk/safeguarding-checklist (accessed 4 March 2021).

We coded all studies that were suitable for quality (risk of bias) assessment, based on use of a recognised design and a corresponding assessment tool. Quality assessments were performed using tools developed by the Joanna Briggs Institute, the CASP tool for qualitative studies and AMSTAR for systematic reviews. Quality assessment was performed by a single reviewer, with a 10% sample checked for accuracy and consistency. Assessment of the overall strength (quality and relevance) of evidence for each research question was incorporated within an accompanying narrative synthesis. The synthesis was descriptive and studies were grouped by intervention type (service development, use of routinely collected data and other) and setting (health care, social care or both).

For studies reporting sufficient details, we used the 5-item TIDieR-Lite checklist (By Whom, What, Where, To What Intensity, How Often) to map intervention components. This modification of the TIDieR framework had been used by the authors in a previous review [2].

### Patient and public involvement

The Sheffield Evidence Synthesis Centre public advisory group was involved throughout the project. In December 2019, the group discussed:

- which groups of health/social care professionals need to be aware of safeguarding children/young people?
- what might be the barriers to awareness and appropriate action?

Group members identified diverse health (particularly allied health) and care professionals in need of safeguarding awareness beyond those covered by studies included in this review. The Group found it challenging to identify barriers, raising the possibility that this question might be more usefully targeted for consultation with professionals.

## Results

### Results of literature search

**Fig. 1:**
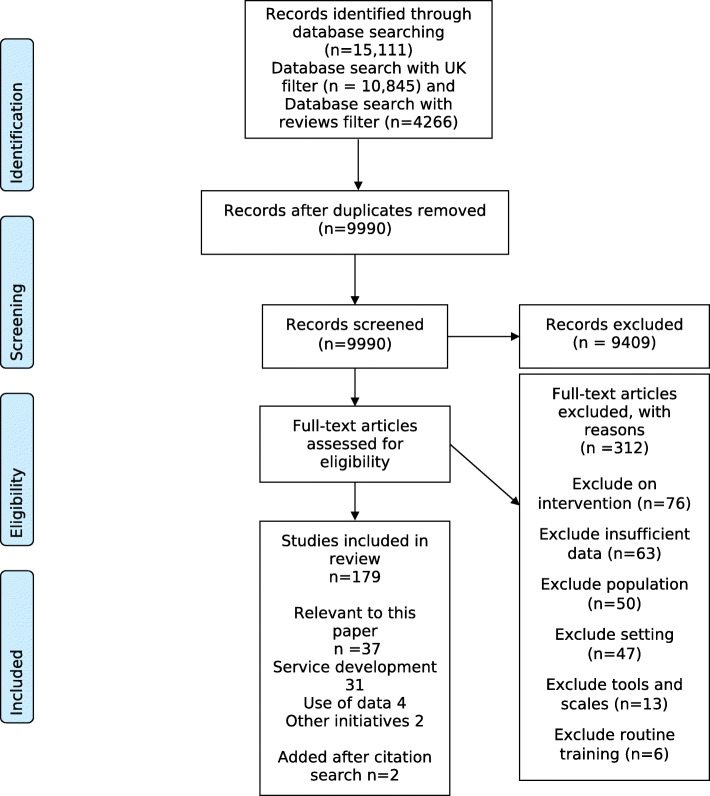
PRISMA flow diagram

The PRISMA flow diagram for the review is presented in Fig. 1.

### Study characteristics and risk of bias

Characteristics of the included studies are summarised in Tables 1, 2, 3 and 4. The majority of studies included in the current analysis used a cross-sectional design, while others were audits or surveys that were not designed as formal research studies. Only one study (two publications) [26, 27] met our criteria for quality assessment. The study lacked a control group and responses to most assessment questions were ‘no’ or unclear, suggesting a high risk of bias (see Appendix 4 of the full report [1]).

**Table 1:** Service development initiatives mainly in health settings

Study	Setting	Professionals involved	Type of service	Type of evaluation	Findings related to awareness
Allnock 2012 [3]	Hospital and community	Multiple groups	Therapeutic services for children who have experienced sexual abuse	Cross-sectional	Significant shortfall in services relative to demand. Identifies need for relevant professionals to be trained to identify vulnerable children
Appleton 2012 [4]	Community	Child protection nurses	Primary care child protection services	Cross-sectional	Challenges include child protection moving off primary care agenda, high threshold for referral to social services
Bajaj 2006 [5]	Hospital	Specialist nurse	Liaison and discharge co-ordinator role	Before/after	Recording and analysis of outcomes can improve understanding of important factors affecting outcomes
Browne 2013 [6]	Community	Health visitors	Family nurse partnership	Cross-sectional	Service can be made most efficient by focusing on families with known risk factors
Care Quality Commission 2009 [7]	Hospital	Multiple groups	Services provided by NHS Trusts	Cross-sectional	Trusts should review safeguarding arrangements and commissioning organisations need to ensure effective safeguarding in general practices
Hodes 2016, 2017 [8, 9] Creighton [10]	Hospital outpatient clinic	Multi-disciplinary team	Clinic for children with known or suspected FGM	Service description and case series	Availability of specialist service in response to awareness and need
Kaye [11]	Hospital ED	ED clinicians	Risk assessment for children of people presenting with mental health problems	Before/after (audits)	Protocol increased awareness of children potentially needing safeguarding
Park 2015 [12]	Community	Dentists	Oral assessment as part of comprehensive medical assessment	Cross-sectional	Oral assessment by a dentist can improve awareness of child protection needs

**Table 2:** Service development initiatives in social care settings

Study	Setting	Professionals involved	Type of service	Type of evaluation	Findings related to awareness
Appleton 2015 [13]	Local authority	Social workers	Strengthening Families child protection conference	Before/after	Most professionals thought approach worked well but families perceived they were being judged
Ashley 2017 [14]	City LSCB area	Social workers and others with safeguarding responsibility	FMEA (Failure Mode and Effects Analysis)	Cross-sectional	FMEA was valuable for participants and generated actions to improve response
Firmin 2016 [15]	Local authorities	Social workers	Contextual social work interventions	N/A (summary of published research)	Interventions that take account of context may improve safeguarding
Gupta 2010 [16]	Social care system	Social workers and other practitioners	Improved recognition and safeguarding of trafficked children	Review of research and cross-sectional (interviews)	Need for improved training and deployment of staff, better interprofessional working and collection and sharing of data
Harris 2017 [17]	Voluntary sector child sexual exploitation (CSE) services	Multiple groups Child protection professionals and CSE workers	‘Hub and spoke’ model, including training for professionals	Cross-sectional	Hub and spoke model improves standards in local safeguarding by extending the reach of training and resources
Heikkila 2011 [18]	Social care system (UK and other European countries)	Social workers and police	Examples of police and social workers working together, including school safety initiatives	Cross-sectional	Shows importance of networks between practitioners and multicultural skills
Hurley 2015 [19]	Social care system	Social workers and others working with Romanian children	International Multi Agency Assessment Framework (IMAAF), a tool to prompt professionals to consider safeguarding issues related to trafficking	Evaluation of the IMAAF was in progress at the time of the report.	IMAAF encourages agencies to work together within and between countries to safeguard trafficked children
Peckover 2017 [20]	Local authorities	Multiple groups Practitioners working in domestic abuse and safeguarding	Development of multiagency working in domestic abuse and child safeguarding	Cross-sectional	Need for further improvement in multiagency working to safeguard children
Pinkerton 2015 [21]	Health & Social Care Trusts in Northern Ireland	Multiple groups Agencies dealing with ‘looked after’ children	Review of cases of ‘looked after’ children who had repeatedly ‘gone missing’ and were at risk of sexual exploitation	Cross-sectional	Improved awareness of ‘going missing’ as a possible indicator of sexual exploitation needing a multiagency response
Whiting 2008 [22]	Local authority	Multiple groups Nurses, health visitors (including ‘health specialists’), social workers and managers	Health specialist initiative (health visitors seconded to child protection teams)	Cross-sectional	The health specialist was successful in improving communication, increasing social workers’ knowledge of child health and strengthening assessments made in social care.

**Table 3:** Service development initiatives spanning health and social care

Study	Setting	Professionals involved	Type of service	Type of evaluation	Findings related to awareness
Bunn 2013 [23]	Health and social care services in England	Multiple groups Multidisciplinary teams	Signs of Safety model for risk assessment and safety planning	Cross-sectional (survey and interviews)	Local authorities using the model in different ways, need for long-term evaluation of outcomes
Care Quality Commission 2016 [24]	Health and social care services in England	Multiple groups	Services for ‘looked after’ children	Cross-sectional	Examples of good and innovative practice but more needs to be done to identify children at risk of harm
Daniel 2010 [25]	Health and social care services in England	Multiple groups Multidisciplinary groups of practitioners from all key professions working with children	Action on Neglect educational resource	Cross-sectional	Availability of support and services in response to early signs of problems will often enable parents to provide required care
Devine 2015 [26, 27]	Health and social care services in England	Multiple groups	Analysis of trends in assessment and referral	Time series	Trend to increased referral but not increased detection of abuse; possible lower threshold for referral
Fifield 2011 [28]	Health and social care in an area of NW England	Multiple groups Multidisciplinary teams Managers	Pilot integrated model involving safeguarding nurses	Cross-sectional (questionnaires)	Model achieved its aim but efficiency was reduced by lack of an integrated IT system
Haynes 2015 [29]	Health and social care services in England	Multiple groups Early years practitioners Health visitors Midwives Schools nurses Teachers; GPs	Services for children at risk of neglect	Cross-sectional (interviews, focus groups and surveys)	Shortfalls in services identified, all practitioners have a role in identifying and providing early help for children suffering neglect
Kaur 2018 [30]	Five local authorities in England	Multiple groups Commissioners, commissioning partners, service providers and local practitioner experts	Commissioned services to address child sexual abuse and exploitation (CSA and CSE)	Cross-sectional	Local authority partnerships are running well-developed CSE initiatives; CSA and harmful sexual behaviour should be targeted with the same rigour as CSE. Health bodies have a role in addressing all three types of abuse.
Spencer 2019 [31]	Dental hospital and local child protection services	Hospital nurse	Paediatric liaison nurse service	Case series with comparison group	Service promotes integrated multidisciplinary working and helps overcome barriers to dentistry’s involvement in safeguarding children.
Webber 2013 [32]	London borough: adult mental health and children’s social care	Multiple groups Social workers (52%); managers; nurses; psychiatrists; clinical psychologists; and occupational therapists	Joint protocols to support multiagency working	Cross-sectional (survey)	Practitioners perceived that the protocols had increased awareness of the risk factors for safeguarding children.

**Table 4:** Initiatives involving use of data

Study	Setting	Professionals involved	Type of initiative	Type of evaluation	Findings related to awareness
Arai 2015 [33]	NHS in England	Multiple groups Interview subjects included service managers; health visitor; safeguarding nurse; consultant paediatricians; and an administrator	Guidelines to follow up non-attendance	Cross-sectional (mapping and interviews)	Better use of non-attendance data could improve awareness of safeguarding concerns
Kirby 2019 [34]	Community dental service in Sheffield	Dental team members	Pathway to follow up children’s missed dental appointments	Retrospective service evaluation and interviews	Missed appointments could indicate neglect, possible need to share information for safeguarding
McGough 2006 [35]	Integrated sexual health service in Glasgow	Multidisciplinary team Staff providing sexual and reproductive health service at a centre that also provides counselling, information and support services.	Recording of data from consultations with clients aged under 16	Case series	Answers to some questions may raise awareness of child protection issues
McGovern 2015 [36]	Eleven general practices in England	GPs	Coding to improve recording of child maltreatment concerns	Before/after (audit)	Improved recording could improve data sharing and identification of children at risk
Mitchell 2019 [37]	Seven hospitals in East Anglia	Paediatricians	Assessment of children with fractures in the ED for risk of physical abuse	Cross-sectional	Detection of possible abuse could be improved by reducing variation in referral to paediatric assessment
Nuttall 2020 [38]	Emergency departments in Bristol, Cardiff and Manchester	ED staff and health visitors	Potential sharing of HV records with ED staff	Prospective, cross-sectional	Data sharing could increase awareness of risk factors in the ED

### Service development

The 31 included papers in this group were divided almost equally between health settings (11 papers), social care settings (10) and services integrated across both systems (10). Table 1 summarises papers dealing primarily with the NHS. Two included papers provide overviews of safeguarding in the NHS [7] and of therapeutic services for children who have experienced sexual abuse [3]. Both studies identified areas for improvement in awareness and safeguarding practice. Similarly, interviews with child protection nurses identified pressures in primary care that could reduce the ability of the health system to respond to child protection needs [4]. These studies were published in 2009 to 2012 so may not fully reflect the current situation. Tompsett et al. noted the existence of conflicts around involvement of GPs in child protection and safeguarding, some GPs seeing their role as primarily referral to social services while other stakeholders anticipated a higher degree of involvement [39].

Other papers report specific service development initiatives within primary care or hospital settings. Studies show that specialist health visitors [6] and dentists performing a comprehensive oral assessment [12] have the potential to contribute to improved awareness and assessment of child protection needs. In the hospital setting, a nurse child protection co-ordinator improved the referral process [5] and an outpatient clinic was established to meet the needs of children with suspected female genital mutilation (FGM) [8, 9]. Finally, Kaye et al. developed a process for increasing awareness of risks associated with parental mental illness and ensuring that children of those presenting with mental illness are assessed for risk and safeguarded as necessary [11].

Ten papers (Table 2) focused on initiatives classified as social care (mainly services provided by local authorities or the voluntary sector, rather than the NHS). These papers described and/or evaluated methods [13–15], service models [17, 22] and initiatives aimed at safeguarding specific groups such as trafficked children or those in local authority care [16, 18–21]. The papers mainly reported cross-sectional evaluations based on qualitative interviews and/or document reviews. Some initiatives appeared promising [14, 17, 22] but problems were also identified, particularly difficulties across agencies with different priorities and world views when working together to improve safeguarding [16, 20].

The ten papers that spanned health and social care (Table 3) reflected similar themes to those from social care. Promising initiatives to promote awareness included local authority partnership child sexual exploitation services (though other related services worked less well) [30]; joint protocols between adult mental health and children’s social services [32]; and a paediatric dentistry liaison service [31] based in a hospital but working between community and social services. In contrast to these positive local examples, studies with a national focus often identified deficiencies in the availability of services and/or training [24, 29] or variations in the delivery of a specific intervention [23]. In one study, integrated working between health and social services was hampered by a lack of compatible record systems [28].

As before, most evaluations in this group were cross-sectional and based on interviews or survey responses rather than numerical data. One exception used long-term data from 1989 onwards to analyse trends in assessment and referral [26]. Only one group of authors included a comparison group, within a study that included routine data on a small number of patients [31].

### Use of data

Six included studies (Table 4) documented initiatives involving use of routine data to improve awareness of safeguarding at the system level in health and/or social care [33, 35–37]. Studies in primary care settings (a sexual health clinic [35] and several general practices [36]) suggested that it is possible to improve data collection in clinical practice to improve identification of possible safeguarding issues. A community dental service developed and evaluated a pathway to follow up missed appointments and share information with other professionals if necessary [34]. The pathway supported early and consistent sharing of information and improved dental team confidence. The two studies conducted in hospitals revealed variation in the handling of missed appointments [33] and in procedures for referring young children with fractures for paediatric assessment [37]. Although a limited sample, these studies suggest that reduction in variation between hospitals may represent one way of improving use of data that are collected routinely and thus improving outcomes for children experiencing or at risk of abuse.

The most recent study investigated sharing of data between health visitors and emergency department (ED) staff in relation to children under 5 years old attending with burns [38]. This prospective multicentre study found that 59% of children with burns lived in families with risk factors for maltreatment. Many risk factors noted on health visitors’ records were not recorded by ED staff despite being part of a standard form. The study authors concluded that sharing of records between community (health visitor) and acute (ED) services would improve awareness and assessment of safeguarding risks.

### Other interventions

Only two studies reported other initiatives [40, 41]. One qualitative study explored reporting of possible abuse by primary healthcare professionals [40]. The other study looked at how cases of child neglect are managed over time and concluded that a new approach is needed, involving collection of evidence that could be used in care proceedings if necessary [41].

### Component analysis

Ten included studies were classified as suitable for component analysis using the TiDIER-Lite checklist: comprising seven studies (eight papers) on service development and three studies on use of data.

### Service development

The eight service development interventions suitable for component analysis (Appendix 1, Supplementary Table 1) comprised new roles [5, 22, 28, 31], a new service for children with actual or suspected FGM [9, 10]; and two initiatives aimed at safeguarding specific groups (migrant/trafficked children [19] and children attending the ED with fractures [11]). The new roles all involved liaison between health and social care and are staffed by nurses/health visitors. The TIDieR-Lite framework makes it possible to compare similar roles. For example, a liaison role based in an acute hospital [5] requires higher levels of staffing than a similar post based in a dental hospital [22]. All the interventions in this group are relatively high intensity, reflecting the complex needs of the groups being served, and the frequency of intervention is flexible depending on need. For example, Bajaj et al. reported that monthly meetings are held to discuss child protection concerns but a co-ordinator is available for advice on a daily basis [5].

These findings, though based on a small number of studies, suggest that different services may have identified similar needs for service models that help different agencies to work together in safeguarding by promoting joint working and information sharing.

### Use of data

Component analysis was possible for five studies of initiatives involving better use of data (Appendix 1, Supplementary Table 2). All the initiatives involved data collected in clinical settings and hence required processes to be as simple as possible without sacrificing rigour. Three of the studies reported on development and piloting of the data collection instrument [34–36], which would be important when introducing a new procedure into routine clinical practice.

### Evidence of effectiveness, feasibility and acceptability

The nature of the included studies made it difficult to establish evidence of the interventions for raising awareness, let alone longer-term effects on actions to prevent abuse. Interventions were identified as ‘promising’ based mainly on interviews with or surveys of professionals who delivered and/or received them. Interventions supported by relatively stronger evidence from before/after or time series studies were a liaison and discharge co-ordinator role [5]; an ED risk assessment protocol [11]; child protection conferences [13]; and improved data coding in general practice [36]. A case series study of a paediatric liaison nurse service had a comparison group but the main finding concerned its effectiveness in promoting interdisciplinary working [31].

Evidence on feasibility largely identified barriers to the implementation of new interventions in safeguarding. Barriers mainly involved existing pressure on services [4] and difficulties in integrated working between different services and/or professional groups [28, 39]. Cost was rarely identified as a barrier because very few studies reported on cost or resource implications. Acceptability was also rarely highlighted but one study reported that some GPs saw their role in safeguarding as limited to referral to social services and had concerns about more active involvement [39].

## Discussion

### Main findings

This review sought to establish what interventions (other than those based on provision of training or information) have been evaluated for promoting awareness and supporting prevention of harm in safeguarding children and young people in UK health and social care settings. A further objective was to identify evidence on outcomes related to effectiveness, feasibility and acceptability of the interventions. We defined awareness broadly to include the facility of the wider system, not just individuals, to process relevant information and respond appropriately. The majority of included studies covered development of services (including those spanning health and social care), while just four studies explored more effective use of routinely collected data to support safeguarding.

We identified several promising service development initiatives, particularly involving new roles or processes to promote effective working between health and social care [22, 31, 32]. At the same time, interagency working was frequently identified as a challenge to the successful implementation of initiatives [16, 20].

Only four studies explored initiatives involving use of routine data to improve awareness of potential safeguarding risks, for example identifying children who regularly miss scheduled health appointments [33]. Improved recording or coding of data [36] and reduction of variation between institutions [37] appear to be promising approaches.

### Strengths and limitations

A key strength of this review is its focus on interventions and initiatives beyond staff training to raise awareness of safeguarding issues. It includes interventions in health, social care and integrated settings, reflecting the diverse services where safeguarding awareness is required and the diverse professional groups who are involved.

We included studies published between 2004 (date of important legislation affecting safeguarding) and 2020. The included studies demonstrate how the evidence base has evolved over time and allow identification of perennial themes. One limitation of this approach is that older papers are likely to be less relevant to current practice. Our inclusion criteria were also broad, with no restrictions on study design and both quantitative and qualitative studies were included. This allowed us to identify potentially promising interventions that might otherwise have been overlooked or neglected. On the other hand, the weak design of many of the included studies means that further evaluation would be required before considering the interventions for wider implementation.

The review was conducted rapidly by a small team. Methodological strengths include a thorough search, including citation searching, and use of the TIDieR-Lite framework to characterise interventions. Study quality was assessed using standard tools when study design and reporting made this possible. Unfortunately, quality assessment was only possible for one of the included studies (two publications) [26, 27] and the results suggested a high risk of bias.

We used several methods to abbreviate the review process, as appropriate for a rapid mapping review of the relevant literature. Verification of items for inclusion/exclusion was limited to a 10% sample and undertaken retrospectively. Inclusion of items was informally checked by team discussion of uncertainties during later stages of the review. A further methodological short-cut was the use of one checklist (the JBI checklist for quasi-experimental studies) to cover several different study designs. This was not a significant limitation for our study given that so few included studies were suitable for formal quality assessment.

Limitations of the evidence base included lack of long-term follow-up, control groups and data on service-relevant outcomes. This may partly reflect different research cultures between healthcare and social care research. None of the included studies reported on costs or value for money. Limitations in reporting constrained our ability to draw conclusions from the component analysis. There was a particular lack of studies on safeguarding in the transition from adolescence to adulthood.

### Relationship to previous research

We believe this to be the first synthesis of evidence on service development and related interventions aimed at increasing safeguarding awareness in health and social care. Our work also differs from most previous reviews in that it covers the whole range of health and social care settings. The full technical report [1] includes a review of reviews of international evidence on this topic, containing 27 relevant reviews. Many of the reviews deal with safeguarding awareness in specific roles (e.g. school nurse, health visitor, paramedic or GP) or settings (e.g. five reviews covered safeguarding in EDs). Other than these groups, few topics had a significant volume of review-level evidence.

This mapping review is also distinctive in its focus on evidence from the UK. Most research performed in UK settings is of relatively low quality in terms of risk of bias. Higher-level overviews and policy documents produced by government departments, NHS bodies and other stakeholders were included in the full report but few of them included consideration of service development issues [1]. The limited evidence base around safeguarding girls and young women from female genital mutilation was identified as a research priority by the National Institute for Health and Care Excellence (NICE) and was one of the factors underlying the commissioning of this research [1]. This paper extends the information available to decision-makers through the use of systematic searching, quality assessment and component analysis of interventions and initiatives. Despite its UK focus, it may be of interest to decision-makers in other health and social care systems, particularly in the context of efforts to integrate health and social care.

### Implications for service delivery and research

The findings of this review imply that health and social care services wishing to improve awareness of child safeguarding issues may benefit from looking beyond the most apparent measure of high-quality training provision. While safeguarding is relevant to all staff, roles vary between those who are a first point of contact for identifying safeguarding concerns (e.g. A&E staff, dentists), those for whom safeguarding forms a major background to their daily work (e.g. school nurses, health visitors) and those who provide specialist support within a safeguarding pathway. Promising service development initiatives include liaison nurses [5, 31], assessment clinics [10], secondment [22], joint protocols [32], and a ‘hub and spoke’ model [17]. We identified few studies on the use of data but this approach appears promising and analysis of routinely collected data is unlikely to involve significant costs. However, service providers need to consider the legal and ethical acceptability of data recording and ensure protection of confidentiality for service users.

In terms of research, there is a clear need to continue and extend mapping and evaluation of service initiatives beyond previously reported work [42]. Longer-term studies with outcomes relevant to service users are needed. Research intended to support effective safeguarding is likely to require active inter-agency collaboration. Research to optimise the use of routine data to identify children at risk of abuse could involve the development of innovative analytical tools. However, improvements in the quality and consistency of data coding would also be valuable. Safeguarding of older adolescents has also been identified as a research need.

Although not investigated in our review, involvement of children/young people and families/carers is likely to be essential for successful design and implementation of safeguarding interventions. Evaluations should also investigate costs/resource use and barriers to successful implementation at different levels of the health and social care system.

## Supplementary Information

**Additional file 1.**

## Data Availability

Any additional data not included in this report and its supplementary files are available on request. All queries should be submitted to the corresponding author.

## References

[1] Chambers D, Cantrell A, Booth A (2020). Recognition of risk and prevention in safeguarding of children and young people: a mapping review and component analysis of interventions aimed at health and social care professionals in.

[2] Chambers D, Cantrell A, Booth A (2020). Factors that facilitate the implementation of interventions to reduce preventable hospital admissions with a focus on cardiovascular or respiratory conditions: an evidence map and realist synthesis. Health Serv Deliv Res.

[3] Allnock D, Radford L, Bunting L, Price A, Morgan-Klein N, Ellis J, Stafford A (2012). In demand: therapeutic Services for Children and Young People who Have Experienced Sexual Abuse. Child Abuse Rev.

[4] Appleton JV (2012). Delivering safeguarding children services in primary care: responding to national child protection policy. Prim Health Care Res Development.

[5] Bajaj M, Mease RG, Allen K, Dryburgh EH (2006). Safeguarding children-is there a role for a coordinator?. Child Abuse Rev.

[6] Browne KD, Jackson V (2013). Community intervention to prevent child maltreatment in England: evaluating the contribution of the family nurse partnership. J Public Health.

[7] Care Quality Commission (2009). Safeguarding children: a review of arrangements in the NHS for safeguarding children.

[8] Hodes D, Armitage A, Robinson K, Creighton SM (2016). Female genital mutilation in children presenting to a London safeguarding clinic: a case series. Arch Dis Child.

[9] Hodes D, Creighton SM (2017). Setting up a clinic to assess children and young people for female genital mutilation. Arch Disease Childhood Education Pract.

[10] Creighton SM, Dear J, de Campos C, Williams L, Hodes D (2016). Multidisciplinary approach to the management of children with female genital mutilation (FGM) or suspected FGM: service description and case series. BMJ Open.

[11] Kaye P, Taylor C, Barley K, Powell-Chandler A (2009). An emergency department intervention to protect an overlooked group of children at risk of significant harm. Emerg Med J.

[12] Park CM, Welbury R, Herbison J, Cairns A (2015). Establishing comprehensive oral assessments for children with safeguarding concerns. Br Dent J.

[13] Appleton JV, Terlektsi E, Coombes L (2015). Implementing the strengthening families approach to child protection conferences. Br J Soc Work.

[14] Ashley L, Armitage G, Taylor J (2017). Recognising and referring children exposed to domestic abuse: a multiprofessional, proactive systems-based evaluation using a modified failure mode and effects analysis (FMEA). Health Social Care Community.

[15] Firmin C, Warrington C, Pearce J (2016). Sexual exploitation and its impact on developing sexualities and sexual relationships: the need for contextual social work interventions. Br J Soc Work.

[16] Gupta A (1801). How should we safeguard trafficked children?. Community Care.

[17] Harris J (2017). Evaluation of the Alexi Project 'hub and spoke' programme of CSE service development: key messages.

[18] Heikkila E (2011). Working together for better integration: immigrants, police and social work.

[19] Hurley B, John-Baptiste M, Pande S (2015). Free to move, invisible to care: coordination and accountability towards Romanian unaccompanied minors' safety.

[20] Peckover S, Golding B (2017). Domestic abuse and safeguarding children: critical issues for multiagency work. Child Abuse Rev.

[21] Pinkerton J (2015). Getting focused and staying focused: 'looked after children', going missing and child sexual exploitation: a thematic review.

[22] Whiting M, Scammell A, Bifulco A (2008). The health specialist initiative: professionals' views of a partnership initiative between health and social care for child safeguarding. Qual Soc Work.

[23] Bunn A (2013). Signs of Safety in England: an NSPCC commissioned report on the Signs of Safety model in child protection.

[24] Care Quality Commission (2016). Not seen, not heard: a review of the arrangements for child safeguarding and health care for looked after children in England. Newcastle upon Tyne: Care Quality Commission.

[25] Daniel B, Burgess C, Whitfield E, Derbyshire D, Taylor J (2010). Noticing and helping neglected children: messages from action on neglect. Child Abuse Rev.

[26] Devine L, Parker S (2015). Rethinking child protection strategy: learning from trends.

[27] Devine L, Parker S (2015). Child protection and assessment.

[28] Fifield L, Blake S (2011). The early intervention safeguarding nurse pilot: an integrated model of working. Community Practitioner.

[29] Haynes A (2015). Realising the potential: tackling child neglect in universal services.

[30] Kaur K, Christie C (2018). Local commissioning of services addressing child sexual abuse and exploitation in England: a rapid review incorporating findings from five locations.

[31] Spencer C, Zaitoun H, White EJ, Harris JC (2019). Role of the dental hospital-based paediatric liaison nurse in safeguarding children. Br Dent J.

[32] Webber M, McCree C, Angeli P (2013). Inter-agency joint protocols for safeguarding children in social care and adult mental-health agencies: a cross-sectional survey of practitioner experiences. Child and Family Social Work.

[33] Arai L, Stephenson T, Roberts H (2015). The unseen child and safeguarding: 'Did not attend' guidelines in the NHS. Arch Dis Child.

[34] Kirby J, Harris JC (2019). Development and evaluation of a ‘was not brought’ pathway: a team approach to managing children’s missed dental appointments. Br Dent J.

[35] McGough P, Thow C, Butt A, Lamont M, Bigrigg A (2006). Recording what happens in the under-16 consultation. Journal of Family Planning & Reproductive Health Care.

[36] McGovern AP, Woodman J, Allister J, van Vlymen J, Liyanage H, Jones S, Rafi I, de Lusignan S, Gilbert R (2015). A simple clinical coding strategy to improve recording of child maltreatment concerns: an audit study. J Innovation in Health Informatics.

[37] Mitchell PD, Brown R, Wang T, Shah RD, Samworth RJ, Deakin S, Edge P, Hudson I, Hutchinson R, Stohr K, Latimer M, Natarajan R, Qasim S, Rehm A, Sanghrajka A, Tissingh E, Wright GM (2019). Multicentre study of physical abuse and limb fractures in young children in the East Anglia region, UK. Archives of Disease in Childhood.

[38] Nuttall D, Rea D, Bennett CV, Hollen L, Mullen S, Maguire S, Emond A, Kemp A, Deave T (2020). Would shared health visitor and emergency department records improve recognition of child maltreatment within the emergency department? A prospective multicentre study. Child Abuse Rev.

[39] Tompsett H, al E (2009). The child, the family and the GP: tensions and conflicts of interest in safeguarding children.

[40] Agravat P, Singh S (2019). Ross J: "you just know something's not right" - what makes primary healthcare professionals suspect child abuse? A qualitative study. Education for Primary Care.

[41] Farmer E, Lutman E (2014). Working effectively with neglected children and their families: what needs to change?. Child Abuse Rev.

[42] Luckock B, Barlow J, Brown C (2017). Developing innovative models of practice at the interface between the NHS and child and family social work where children living at home are at risk of abuse and neglect: a scoping review. Child and Family Social Work.

