# Supplementary material for: Recognition of risk and prevention in safeguarding of children and young people: a mapping review and component analysis of service development interventions aimed at health and social care professionals
Source: BMC Health Serv Res. 2021 Nov 17;21:1241. doi: 10.1186/s12913-021-07257-8 (PMC8600929; doi:10.1186/s12913-021-07257-8)
Supplement: Supplementary file 1 — Additional file 1. [file 12913_2021_7257_MOESM1_ESM.docx]

# Appendix 1: supplementary tables

Supplementary Table 1: summary of components of service development initiatives

| **Reference** | **By whom** | **What** | **Where** | **To what intensity** | **How often** |
| --- | --- | --- | --- | --- | --- |
| Bajaj 2006 [11] | Children's Liaison and Discharge Co-ordinator | Coordinator raises awareness, training, provides advice, ensuring documentation is completed, investigate and follow-up concern raised and involvement in monthly meetings to review child protection concerns. | Child Protection Team Peterborough District Hospital | Initial post for a year, one full-time coordinator employed and workload meant a second part-time coordinator was employed. Coordinators available 8-5pm for advice, investigate concerns. Monthly meetings to reviews child protections concerns raised. | Monthly meetings to review any child protection concerns raised within the trust but available for advice and to investigate concerns on a daily basis. |
| Creighton 2016[32]; Hodes 2017[13] | Team led by consultant paediatrician with gynaecologist, specialist nurse, psychotherapist. Play specialist support. Independent interpreters and telephone interpreters are available if required. | Genital examination using a colposcope. Where FGM is confirmed, testing for blood-borne viruses (BBV) is recommended. After the consultation, the findings are explained to the parents, social worker and police if present. Children and families are routinely offered a debriefing session with the psychotherapist. A small number of follow-up psychotherapy sessions can be provided in the clinic but children are referred back to local child mental health services for longer term support. | Specialist paediatric outpatient clinic | Dedicated multidisciplinary service reflecting patients' complex needs | Detailed assessment with follow-up if required. |
| Fifield 2011 [31] | Specialist community public health nurse (school nurse or health visitor) | To provide health advice and knowledge for children's services, health input to assessments, and act as an advocate for health visitors and school nurses who had concerns over referral to children's services. | Location not stated but co-located with social workers and managed by social workers' manager. | Full-time position? | Regular daily basis |
| Hurley 2015[21] | Social workers involved in assessing a child's needs beyond immediate protection and welfare. | International Multi Agency Assessment Framework (IMAAF) has three domains which deal with agencies that may be relevant in understanding and assessing the child's needs; establishing the credentials of any adult(s) that may be accompanying the child; and issues in the wider environment that may be relevant to the child's situation. | Social care and related children's services | As required to obtain necessary information and provide an appropriate assessment and services. | One-off assessment with appropriate follow-up. |
| Kaye 2009[14] | Hospital ED staff supported by mental health team | Redesigned mental health proforma with questions about children and their circumstances and prompt to complete 'cause for concern' form; 'cause for concern form specifically for children of parents with mental illness; education programme for all grades of medical and nursing staff. Patients were informed of any referral to social services. | Observation unit associated with ED (most patients stay overnight or a similar length of time before assessment by the psychiatric liaison team) | Intervention aimed to identify and risk stratify all dependent children of patients attending the ED for mental health problems. | One-off intervention on presentation to the ED. |
| Spencer 2019[27] | Paediatric liaison nurse working 3 days per week | Paediatric liaison nursing service, promoting two-way communication between the dental hospital and other health professionals using an agreed pathway. | Service based in dental hospital | Intensity of intervention as required, including child protection referral to social services | As required while child is undergoing dental treatment |
| Whiting 2008[19] | Trained health visitors | Health specialist role, working alongside social workers to undertake joint assessments. | Local authority child welfare teams. | Intervention allows a fuller assessment than would be possible for a social worker alone and ensures health and development issues are fully covered. | As required in the context of individual cases. |

Supplementary Table 2: summary of components of initiatives involving use of routine data

| **Reference** | **By whom** | **What** | **Where** | **To what intensity** | **How often** |
| --- | --- | --- | --- | --- | --- |
| Kirby 2019[35] | Community dental team members | New pathway consisting of three components: explanatory flowchart; templates for clinical notes with prompts for action; and editable template letters | Dental clinic following a missed (was not brought) appointment | Two phone calls to parent followed if necessary by a letter; referral to clinician if no response to letter in 3 weeks | Of 134 WNB appointments, the pathway was followed consistently in 113 (84.3%) |
| McGough 2006[36] | All staff providing clinical consultations for clients aged under 16 years. | Data recording form developed by a small group of doctors and nurses and piloted before full introduction. | Form was completed during clinical consultations at the Sandyford Initiative. | Staff were requested to complete a recording form for each attendance of a client under 16. Forms were completed for 54.6% of all attendances by female clients aged under 16 years (527/965). | The median number of visits per client was one (range, one to seven). Some 53% of clients for whom forms were returned attended only once during the study period. |
| McGovern 2015[37] | GPs, administrative staff or health visitors in participating practices. | The coding strategy was developed in May 2011. Implementation was supported by implementation packs sent to practices (November 2011); and pre-implementation training and dissemination (November-December 2011). Additional e-mail reminders to use the 'cause for concern' code were sent in January 2012. The strategy was implemented throughout 2012, with a teleconference to discuss progress in February 2012. | General practice consultations. | Minimum coding indicated child is/is not cause for concern. Additional codes covered: is the family cause for concern?; child protection/social care services involved?; and what other professionals are involved? | Concerns were recorded at all relevant consultations |
| Mitchell 2019[38] | Paediatrician | Review to assess risk of physical abuse followed by skeletal scan if necessary | Hospital emergency department | Not applicable | Once |
| Nuttall 2020[39] | Emergency department (ED) clinicians | Burns & Scalds Assessment Tool (BaSAT) | ED or minor injuries unit | For cases with risk factors known to health visitors, EDs recorded five of 47 (10.6%) for domestic violence, ten of 45 (22.2%) with social care involvement and four of 23 (17.4%) with developmental impairment. | Once |
